# Mineral and Skeletal Homeostasis Influence the Manner of Bone Loss in Metabolic Osteoporosis due to Calcium-Deprived Diet in Different Sites of Rat Vertebra and Femur

**DOI:** 10.1155/2015/304178

**Published:** 2015-05-04

**Authors:** Marzia Ferretti, Francesco Cavani, Alberto Smargiassi, Laura Roli, Carla Palumbo

**Affiliations:** ^1^Dipartimento di Scienze Biomediche, Metaboliche e Neuroscienze-Sezione di Morfologia Umana, Università di Modena e Reggio Emilia, Istituti Anatomici, 41124 Modena, Italy; ^2^Laboratorio di Patologia Clinica Endocrinologia, Dipartimento Interaziendale ad Attività Integrata Medicina di Laboratorio ed Anatomia Patologica, AUSL Modena, 41126 Modena, Italy

## Abstract

Rats fed calcium-deprived diet develop osteoporosis due to enhanced bone resorption, secondary to parathyroid overactivity resulting from nutritional hypocalcemia. Therefore, rats provide a good experimental animal model for studying bone modelling alterations during biochemical osteoporosis. Three-month-old Sprague-Dawley male rats were divided into 4 groups: (1) baseline, (2) normal diet for 4 weeks, (3) calcium-deprived diet for 4 weeks, and (4) calcium-deprived diet for 4 weeks and concomitant administration of PTH (1-34) 40 *µ*g/Kg/day. Histomorphometrical analyses were made on cortical and trabecular bone of lumbar vertebral body as well as of mid-diaphysis and distal metaphysis of femur. In all rats fed calcium-deprived diet, despite the reduction of trabecular number (due to the maintenance of mineral homeostasis), an intense activity of bone deposition occurs on the surface of the few remaining trabeculae (in answering to mechanical stresses and, consequently, to maintain the skeletal homeostasis). Different responses were detected in different sites of cortical bone, depending on their main function in answering mineral or skeletal homeostasis. This study represents the starting point for work-in-progress researches, with the aim of defining in detail timing and manners of evolution and recovery of biochemical osteoporosis.

## 1. Introduction

Osteoporosis is a systemic skeletal disease that represents a significant public health problem in an increasingly aging society. It is characterized by net bone loss and microarchitectural deterioration of bone tissue, due to an imbalance between the resorption and formation phases in the bone remodelling cycle (increased bone resorption and reduced bone formation), with a consequent increase in bone fragility and susceptibility to fracture [1–10]. Osteoporosis can be mainly due to mechanical disuse or metabolic alterations. According to the latter, it is well known that both estrogen and calcium deficiencies are important risk factors in the pathogenesis of biochemical osteoporosis. Bone development requires adequate amounts of many nutrients and, among these, calcium is the most important mineral element, since the skeleton represents the greatest calcium store in the body [11]. In fact, more than 95% of the body's calcium is present in bone tissue as hydroxyapatite, conferring rigidity, hardness, and structural integrity to the skeleton [12]. Low calcium intake (particularly common in many countries) and important decrease in calcium intestinal absorption are some of the most important causes involved in bone loss in aging population. Diet is a modifiable risk factor for osteoporosis and adequate amounts of calcium are essential lifelong to maintain healthy bone mass [13, 14]. Several investigations have underlined the role of serum calcium variations in etiopathogenesis of osteoporosis and fracture occurrence; low blood calcium level activates PTH which, in turn, stimulates the production in the kidney of vitamin D that enhances the calcium uptake in the active sites of duodenum [15], while PTH decreases the urinary excretion of calcium and stimulates calcium resorption from bone [16].

It is well known that rats fed calcium-deprived diet develop osteoporosis, as a result of exaggerated bone resorption, induced by parathyroid overactivity, secondary to nutritional hypocalcemia [17–25]. Therefore, rats provide a good experimental animal model for studying bone remodelling alterations during biochemical osteoporosis. It has been reported by Shen and coworkers [26] that the bone mineral density in the rat femoral diaphysis was decreased by a calcium-deficient diet but not by ovariectomy. Other authors suggested that rat ovariectomy plus calcium deficiency results in a great decrease of bone volume and cross-sectional area compared to the calcium alone [27]. Similar results were also observed in an investigation of Donahue and coworkers [28] in which calcium-deficient ovariectomized rats showed decreased values of bone density, similar to those of estrogen-deficient osteoporotic women. Hara et al. [29] suggested that the interaction between ovariectomy and calcium-deficient diet implies different bone alterations in rats, depending on different skeletal segments and/or different skeletal regions with different metabolism. Moreover, dietary calcium deficiency, regardless of ovariectomy, induced bone loss and increased bone turnover in some skeletal segments, in particular in the hard palate, mandible, and proximal tibia. It is interesting to underline that when OVX rats were fed low calcium diet, the decrease in calcium absorption became more significant and resulting bone loss was particularly enhanced [30–32].

The aim of the present investigation was to study how the mineral and skeletal homeostasis influence the bone loss in metabolic osteoporosis due to calcium-deprived diet in different sites of the two bony architectures (trabecular versus cortical bone) in both axial and appendicular skeleton. This model seems to be a good starting point for successive studies on bone alterations during unbalanced calcium metabolism, frequently occurring in aging, with the final goal of defining timing and manners of bone mass recovery when calcium is restored in the diet and verifying the eventual differences of recovery between compact and spongy bones (i.e., the two bone architectures with different metabolism and target).

## 2. Materials and Methods

### 2.1. Experimental Animals and Treatment

Eighteen 3-month-old Sprague-Dawley male rats were purchased from Charles River Laboratories (Calco, Lecco, Italy). All rats were housed individually in single cages, to better check food intake of each rat, and maintained under laboratory controlled conditions (22 ± 1°C, 55–60% humidity, 12 h light : 12 h dark). After 7 days of acclimation to housing conditions, the rats were randomized into four groups, indicated as follows:

 Group 1 (baseline, *n* = 3): sacrificed after 7 days of acclimation;
 Group 2 (control, *n* = 5): fed normal diet for 4 weeks;
 Group 3 (*n* = 5): fed calcium-deprived diet and distilled water* ad libitum* for 4 weeks;
 Group 4 (*n* = 5): fed calcium-deprived diet and distilled water* ad libitum*, plus concomitant administration of PTH (1-34) 40 *μ*g/kg/day, for 4 weeks.

Both normal and calcium-deprived diets were provided daily; at different times during the test period, the food container was briefly removed and weighed to determine quantity of food consumed.

The calcium-deprived diet is a casein based synthetic diet containing a very low amount of calcium (0.04% Ca). PTH (1-34) was supplied by Eli Lilly and Company (Indianapolis, USA), solubilised in saline (40 *μ*g/mL), and subcutaneously injected in a volume of 100 *μ*L/100 gr body weight per rat.

Group 1 animals underwent a subcutaneous injection of calcein (Fluka, St. Louis, MO, USA) 15 mg/kg, two days before the sacrifice. Groups 2, 3, and 4 animals received a subcutaneous injection of calcein 15 mg/kg on the first day of the experimental period, a subcutaneous injection of oxytetracycline hydrochloride (Sigma, St. Louis, MO, USA) 30 mg/kg after 20 days, and a subcutaneous injection of Alizarin-Red S (Fluka, St. Louis, MO, USA) 30 mg/kg 2 days before sacrifice, in order to evaluate newly formed bone during animal treatment.

The body weight of each animal was recorded at the time of arrival in the housing facility and before euthanasia. At the end of the treatment, all rats were anesthetized with ether and blood samples were collected by cardiac puncture; then, rats were euthanized by exsanguination under ether anesthesia.

All experiments were carried out according to the Bioethical Committee of the Italian National Institute of Health. Animal care, maintenance, and surgery were conducted in accordance with Italian law (D.L. number 116/1992) and European legislation (EEC number 86/609).

### 2.2. Histology and Histomorphometry

Soon after euthanasia, the fifth lumbar vertebra (L5) and the right femur of each animal were removed, deprived of soft tissues, fixed in sodium phosphate-buffered (PBS) 4% paraformaldehyde pH 7.4, dehydrated in graded ethanol, and embedded in methyl-methacrylate resin (Sigma Aldrich, Milan, Italy). The vertebrae and the femurs were transversally cut with a Leica SP 1600 diamond saw microtome cutting system (Leica SpA, Milan, Italy) to obtain serial 200 *μ*m thick sections. The sections, taken from the central level of the lumbar vertebral body and from the femur mid-diaphysis and distal metaphysis (for this last considering the more proximal section of the patellar grove), were glued to a glass slide and ground to a final thickness of about 40 *μ*m. These sections were superficially stained with Alizarin-Red and scanned with Epson 3200 perfection scanner at 3200 dpi resolution to perform histomorphometry by means of the software Image J (NIH, Bethesda, USA). The following* static histomorphometric parameters* were calculated: trabecular bone volume (BV/TV), trabecular thickness (Tb.Th), trabecular number (Tb.N), and trabecular separation (Tb.Sp) of the vertebral bodies and the femoral distal metaphyses; the cortical bone thickness (Ct.Th) of the anterolateral and posterior sides of vertebral bodies; the total cross section area, the cortical bone area (Ct-B-Ar), and the medullary canal area of the femoral middiaphyses; the cortical bone area (Ct-B-Ar) of the femoral metaphyses.

The same sections were also further polished to remove the Alizarin-Red staining and used for evaluation of dynamic histomorphometry (by means of bone labelling technique), as a measure of newly formed bone during animal treatment, that is, to distinguish preexistent bone with respect to the bone formed during the period of administration of the calcium-deprived diet. The sections were photographed using a Nikon Eclipse 90i microscope (Tokyo, Japan) equipped with a DS-Fi1 Nikon digital camera and driven by the Nikon ACT-2U software;* dynamic histomorphometric parameters* were evaluated by means of the image analysis system software Image J (NIH, Bethesda, USA). The newly formed bone area (Nf B Ar) and the mineral apposition rate (MAR) were measured between the first two labels (calcein and oxytetracycline) on the anterior and posterior sides of the vertebral cortical bone as well as on the femoral mid-diaphysis. Moreover, the mineralizing surface (MS), marked with Alizarin, was measured on the femoral mid-diaphyses as well as on trabecular bone of vertebral bodies and femur metaphyses. MAR at the femur metaphyseal level was measured taking into consideration the second and third label (oxytetracycline and alizarin). All measurements were performed according to the ASBMR histomorphometry nomenclature [33].

In order to evaluate the presence of osteoid seams in trabecular bone of both femur metaphyses and vertebral bodies, one section (200 *μ*m thick) adjacent to those used for histomorphometry was glued to a metacrylate support, cut to obtain 5 *μ*m thick sections (Reichert-Jung 1150/Autocut), and stained with Gomori trichrome. The osteoid surface (OS/BS) was measured on trabecular bone.

### 2.3. Serum Biochemical Analysis

Blood samples were centrifuged to separate serum that was preserved in tubes, immediately separated by centrifugation (4°C) at 1,500 g for 15 min. Sera were then aliquoted into small volumes and stored at −20°C for successive analyses. The levels of total calcium (Ca) and inorganic phosphorus (P) in serum were determined using the high performance Beckman Coulter analyzer AU680 Chemistry System. The immune-metric assays for the determination of levels of osteoprotegerin (OPG), specific bone alkaline phosphatase (BALP), CTX (Beta CrossLaps), and bioactive-intact-PTH (1-84) in rat serum were provided by Pantec s.r.l. (Turin, Italy); all kits are intended for research use only. In particular, rat-OPG and rat-BALP are two ELISA kits produced by SunRed Hotecnology Company (Shanghai, China); RatLaps is an EIA kit produced by Immunodiagnostic Systems Ltd. (Boldon, UK); rat bioactive-intact-PTH is an ELISA method produced by Immunotopics Inc. (San Clemente, CA). The small amounts of reagents supplied in the kits prevented the possibility to perform automated procedures on laboratory analytical platform. To minimize the variables influencing the test, all good laboratory practice principles were applied: immediate storage of samples after serum separation at −20°C; manual execution of tests in close agreement to the manufacturer's instructions; execution of all tests over two consecutive days in order to avoid repeated freeze/thaw cycles of samples.

### 2.4. Statistical Analysis

One-way analysis of variance (ANOVA) with Bonferroni test between treatment groups and controls was performed using the Software STATA 11.0 (StataCorp, Texas, USA). Values of *P* < 0.05 indicate significant differences between groups.

## 3. Results

### 3.1. Body Weight

Table 1 reports mean values of body weights of all groups recorded both at the time of arrival in the housing facility and at sacrifice; no significant differences were found among groups.

### 3.2. Histology and Histomorphometry

The observations reported in the present paper refer to morphological and histomorphometric evaluations performed on transverse sections of the 5th lumbar vertebra and right femur at middiaphyseal and distal metaphyseal levels of all rats.

#### 3.2.1. Vertebra (L5)

**Figure 1:**
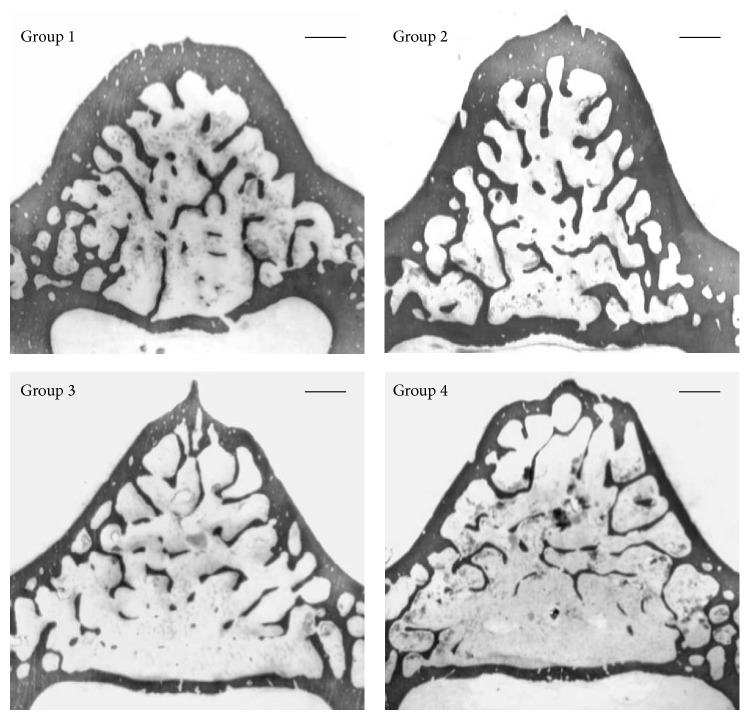
Scans showing bone histology of the transversal sections of the 5th lumbar vertebral body of all animal groups. Group 1: baseline; Group 2: control, normal diet; Group 3: calcium-deprived diet for 4 weeks; Group 4: calcium-deprived diet, plus concomitant administration of PTH (1-34) 40 *μ*g/kg/day, for 4 weeks. Scale bar: 500 *μ*m.

Concerning the vertebral sections, in rats fed calcium-deprived diet, as expected, bone trabeculae are less abundant and cortical bone appears less thick with respect to rats fed normal diet (Figure 1).

Table 2 shows the mean values of the trabecular bone volume (BV/TV), the trabecular thickness (Tb.Th), the trabecular number (Tb.N), and the trabecular separation (Tb.Sp). The results are in line with the morphological observations in showing a statistically significant decrement of trabecular bone of the vertebral body in rats fed calcium-deprived diet (Groups 3 and 4), with respect to rats fed normal diet (Groups 1 and 2). In fact, significant lower values of BV/TV and Tb.N are observed in both groups of rats fed calcium-deprived diet with respect to rats fed normal diet. Similar results were recorded for Tb.Sp, since significant higher distance among bony trabeculae was recorded in both groups of rats fed calcium-deprived diet with respect to rats fed normal diet. As far as trabecular thickness is concerned, instead, significant lower values of Tb.Th were recorded only in rats fed calcium-deprived diet plus PTH (1-34) administration (Group 4) with respect to baseline rats (Group 1).

The results of cortical bone thickness (Ct.Th) in anterolateral as well as in posterior sides of the vertebral body are shown in Table 3: in the anterolateral side, the mean values are significantly lower in both groups of rats fed a calcium-deprived diet than in the rats fed a normal diet; otherwise, in vertebral posterior side the mean values are similar in all groups.

**Figure 2:**
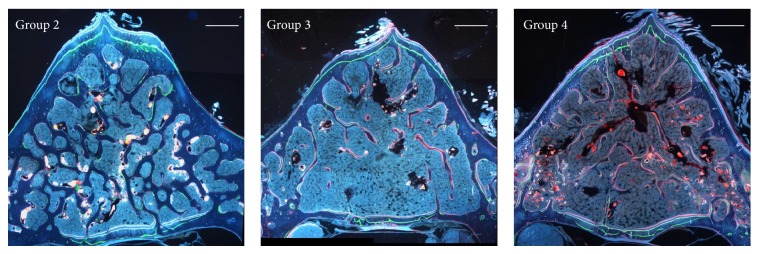
Fluorescence microscope micrographs showing transverse sections of the 5th lumbar vertebral body. Note the newly formed bone among three labels in the anterior and posterior cortical bone and the red fluorescence mostly on the surface of the few trabecular remnants of Groups 3 and 4. Group 2: control, normal diet; Group 3: calcium-deprived diet for 4 weeks; Group 4: calcium-deprived diet, plus concomitant administration of PTH (1-34) 40 *μ*g/kg/day, for 4 weeks. Scale bar: 500 *μ*m.

Figure 2 shows histological transverse sections of the vertebral body from each animal group, under fluorescence microscope. The control group as well as the two groups fed calcium-free diet shows different locations of the labels of osteogenesis: concerning the anterior cortex the labels are observed at periosteal level, while in the posterior cortex the labels are located at the endosteal level. Moreover, in the two calcium-deprived diet groups, the endosteal surface of the anterior cortex shows a thinner layer of bone between the calcein label (injected at the beginning of the experiment) and the bone marrow surface, with respect to control group.

The results of dynamic histomorphometry show that both in the anterior and posterior cortical bone the mean values of Nf B Ar and of MAR are always similar in all groups (Table 4). The trabecular bone shows intense red fluorescence (alizarin) mostly located on the surface of the few trabecular remnants of the groups fed calcium-deprived diet, being instead scarcely present in the animals fed normal diet (Figure 2: Groups 3-4 versus Group 2). Table 4 also shows the mean values of MS of trabecular bone; the results are in line with the morphological observations in showing a statistically significant increase of mineralization surface in the two groups fed calcium-deprived diet with respect to the control one.

Gomori trichrome stain performed on vertebral trabecular bone does not reveal the presence of osteoid seams (OS/BS) in all rats of the control group, while the value of OS/BS in all rat fed calcium-deprived diet ranges from 0 to 24%.

#### 3.2.2. Femoral Mid-Diaphysis

**Figure 3:**
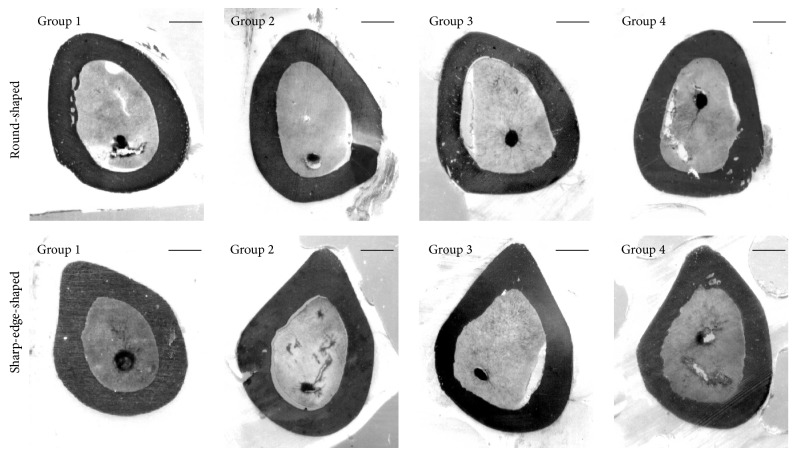
Scans showing bone histology of the transversal sections of the femoral mid-diaphysis of all animal groups. Group 1: baseline; Group 2: control, normal diet; Group 3: calcium-deprived diet for 4 weeks; Group 4: calcium-deprived diet, plus concomitant administration of PTH (1-34) 40 *μ*g/kg/day, for 4 weeks. Scale bar: 750 *μ*m.

Figure 3 shows the middiaphyseal femoral sections of all animals. In each group, among all sections observed, two different morphologies were identified: (i) the more distal mid-diaphyseal sections show oval appearance; thus they were named “round-shaped” sections; (ii) the more proximal mid-diaphyseal sections show sharp-edge appearance (corresponding to the trochanter tertius); thus they were named “sharp-edge-shaped” sections.

Static histomorphometric parameters are reported in Table 5. Mean values of total cross section area and cortical bone area were similar in all groups both in* round-shaped* and in* sharp-edge-shaped* sections. The medullary canal areas are instead always larger in the rats fed calcium-deprived diets (Group 3) with respect to baseline and control ones in both* round-shaped* and* sharp-edge-shaped* sections; this difference is sometimes significant.

**Figure 4:**
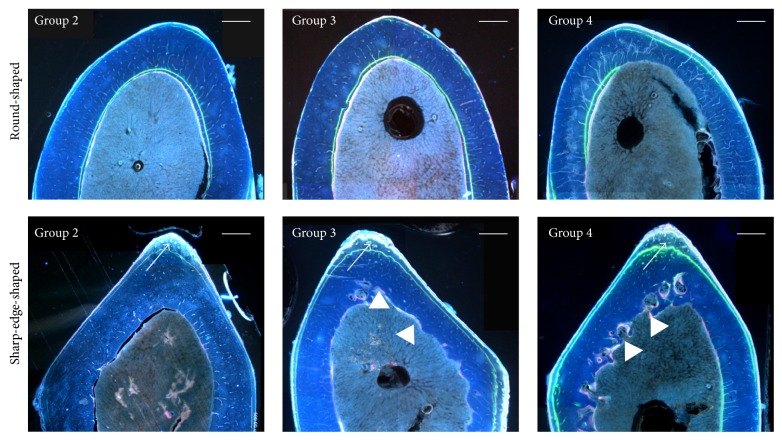
Fluorescence microscope micrographs showing a portion of transverse sections of the femoral mid-diaphysis. Note in* sharp-edge-shaped* sections the new bone deposition mostly located at the periosteal surface (arrows) and bone resorption at the endosteal surface (arrow heads). Group 2: control, normal diet; Group 3: calcium-deprived diet for 4 weeks; Group 4: calcium-deprived diet, plus concomitant administration of PTH (1-34) 40 *μ*g/kg/day, for 4 weeks. Scale bar: 500 *μ*m.

Figure 4 shows, for each animal group, details of histological transverse sections of the femur mid-diaphysis observed under fluorescence microscope. Bone labeling (index of bone osteogenesis) is present mainly at the periosteal surface, in particular in the* sharp-edge-shaped* sections in all groups; moreover, again in particular in* sharp-edge-shaped* sections, the endosteal surface shows an irregular outline (index of bone resorption) in all animals fed a calcium-deprived diet.

The results of dynamic histomorphometry concerning Nf B Ar, MAR, and MS are shown in Table 6: no significant differences were observed among all groups in both* round-shaped* and* sharp-edge-shaped* sections at the periosteal and endosteal level. It must be underlined that in four animals out of five in the two groups fed a calcium-deprived diet and in two out of five animals in the control group we never observed new bone formation at the endosteal level in* sharp-edge-shaped* sections; this implies high values of standard deviation of Nf B Ar and MAR average.

#### 3.2.3. Femoral Metaphysis

**Figure 5:**
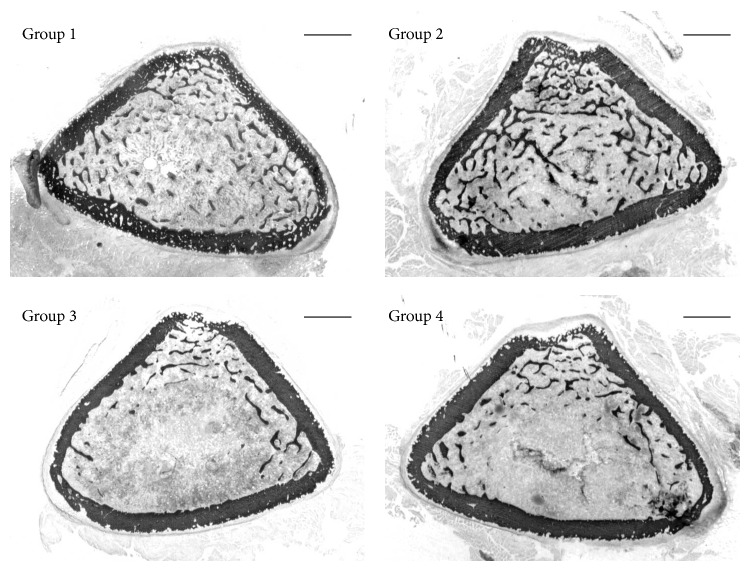
Scans showing bone histology in the transversal sections of the femoral distal metaphysis of all animal groups. Group 1: baseline; Group 2: control, normal diet; Group 3: calcium-deprived diet for 4 weeks; Group 4: calcium-deprived diet, plus concomitant administration of PTH (1-34) 40 *μ*g/kg/day, for 4 weeks. Scale bar: 1 mm.

Figure 5 shows lower amount of trabecular bone in all animals fed a calcium-deprived diet with respect to basal and control ones.

Table 7 concerning static histomorphometry values shows (a) significant lower values of BV/TV and Tb.N in Groups 3 and 4 compared to Groups 1 and 2; (b) significantly increased Tb.Sp values in Groups 3 and 4 compared to Groups 1 and 2; (c) no differences among all groups for Tb.Th and Ct-B-Ar.

**Figure 6:**
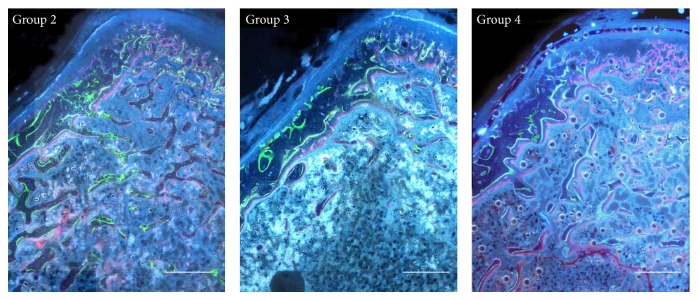
Fluorescence microscope micrographs showing portions of the transverse sections of the femoral distal metaphysis. New bone deposition is present only at the endosteal level and around some trabeculae. Group 2: control, normal diet; Group 3: calcium-deprived diet for 4 weeks; Group 4: calcium-deprived diet, plus concomitant administration of PTH (1-34) 40 *μ*g/kg/day, for 4 weeks. Scale bar: 500 *μ*m.

Figure 6 shows details of histological transverse sections of the femur metaphysis, observed under fluorescence microscope. New bone deposition is observed only at the endosteal level and around some trabeculae in all animal groups. Data reported in Table 8 confirm such histological observations; in particular, the only significant difference is recorded in MS that is higher in rats fed calcium-deprived diet plus PTH (1-34) with respect to control ones.

Gomori trichrome stain does not reveal the presence of osteoid seams (OS/BS) in all rats of the control group, while the value of OS/BS in all calcium-deprived diet animals ranges between 0 and 22%.

### 3.3. Serum Biochemical Analysis

In Table 9 are reported, for each rat, the values of parameters from sera collected at the end of experiment. The values of Ca, P, OPG, and BALP do not show individual variability among the animals inside each group; moreover, the mean values do not show statistically significant differences among the various groups. Concerning, instead, the values of CrossLaps and PTH (1-84) the wide individual variability among the animals inside each group is to be noticed; also for these parameters, no differences were recorded in the mean values among all groups.

## 4. Discussion

The present investigation analyses how the mineral and skeletal homeostasis influence the bone loss in metabolic osteoporosis due to calcium-deprived diet in different sites of the two bony architectures (trabecular versus cortical bone) in both axial and appendicular skeleton.

The first point to discuss concerns the different amount of bone mass recorded between the control group and the two groups of rats fed calcium-deprived diet; in fact, trabecular bone of vertebral bodies and femoral metaphyses (Tables 2 and 7) as well as cortical bone of the vertebral anterolateral part only (Table 3) are significantly reduced in rats fed calcium-deprived diet. Such bone reduction indicates that calcium-deprived diet induces marked bone resorption on those specific bone areas, mainly devoted in answering metabolic demands, according to the well-known mineral homeostasis, as also reported by other authors [26, 29, 30, 34, 35]. In all rats fed calcium-deprived diet, despite the significant reduction of trabecular bone volume and number, the remaining few trabeculae show active sites of bone deposition, demonstrated by the intense red fluorescence on their surfaces as well as by high MS values (Tables 4 and 8); this evidence does not occur in control group. Similar results were observed in OS/BS values, showing that new osteoid is deposited around the few trabecular remnants in calcium-deprived diet rats only. These findings might explain the fact that trabecular thickness is similar in all groups. Altogether these observations agree in suggesting that in all rats fed calcium-deprived diet bone deposition around the remaining few bony trabeculae occurs in answering mechanical demands, according to skeletal homeostasis. On the other hand, in control rats, since no metabolic alterations are observed and no bone mass loss occurs, skeletal homeostasis is not altered, the MS value is very low, and the osteoid secretion (inferable by OS/BS) is absent. Such consideration is particularly true for vertebral bodies trabeculae, whereas in femoral metaphyses the MS values show minor differences between the control group and the groups fed calcium-deprived diet (the difference reaches statistical significance only in the group treated with Teriparatide versus control group). Moreover, at both femoral and vertebral levels, it is interesting to note that OS/BS values are at least half the values of MS in calcium-deprived rats; this finding is probably due to a high osteoid mineralization rate. Our results are in line with those of Shin and coworkers [36] that found in rats (4 weeks after ovariectomy) reduction of BV/TV and Tb N, as well as increase of Tb.Sp of both 4th lumbar vertebra and femur. They also report that, 8 weeks after ovariectomy, further increase of Tb.Sp mainly depends on trabecular thinning at vertebral level and on trabecular number decrease at femoral level. These last results matched with the findings of Thompson et al. [37] that observed after 8 weeks from ovariectomy that rat vertebrae lost bone by thinning trabeculae, whereas proximal tibiae lost bone by removing trabeculae.

The second point to discuss is that the anterolateral part of vertebral cortical bone shows intense modelling processes in all animal groups. In fact, independently of diet type, new bone deposition always occurs at the periosteal level, as shown by the presence of the 3 labels of osteogenesis in all groups (Figure 2); thus, the lack of calcium in diet does not alter the amount of newly formed bone (Table 4). In all rats fed calcium-deprived diet only, intense resorption activity occurs at the endosteal level that leads to the thinning of cortical bone, as also shown by the small amount of bone observed between the calcein label (injected at the beginning of the experiment) and the bone marrow surface. These histological and histomorphometrical data suggest that new bone deposition at the periosteal level likely depends on mechanical needs, whereas bone resorption at the endosteal level likely depends on metabolic demands. Moreover, in all groups the posterior part of vertebral cortical bone shows modeling processes due to new bone deposition at the endosteal level, in answering mechanical needs. These results suggest that cortical bone of the anterolateral side is mainly involved in mineral homeostasis with respect to the posterior one; in fact, the thickness of the anterolateral side of the cortex is significantly lower in rats fed calcium-deprived diet with respect to the control ones, while the thickness of the posterior side is similar in all groups.

As regards the femoral mid-diaphysis, the lack of calcium in the diet leads to an enlargement of the medullary canal due to resorption at the endosteal level, in particular in* sharp-edge-shaped* sections (Figure 3 and Table 5). The finding that only the medullary canal area shows differences among groups, whereas the cortical bone area does not, likely depends on the amount of resorbed bone that is irrelevant when compared to the cortical bone area; this notwithstanding such difference is significant when compared to the medullary canal area. In* sharp-edge-shaped* sections (Figure 4) the medullary canal area increases in all animals fed calcium-deprived diet due to bone modelling that implies a marked periosteal bone deposition at the tertius trochanter (where mechanical loads are greater) and a significant endosteal bone erosion (where mechanical loads are lesser). Dynamic histomorphometric results show that (i) at the periosteal level the amount of newly formed bone is similar in all groups; (ii) at the endosteal level in* sharp-edge-shaped* sections newly formed bone deposition is virtually absent in animals fed calcium-deprived diet (Table 6) and the morphology of endosteal surface is irregular since bone resorption occurs. These observations are in line with data shown in literature [38] reporting that the pattern of cortical bone loss in osteoporosis begins from the endosteal surface of the cortex, where the enlargement of medullary canal occurs at the expense of the cortex inner side; bone loss usually does not occur at the periosteal surface.

In femoral cortex metaphysis, the values of Ct-B-Ar and endosteal MAR do not show differences among groups (Tables 7 and 8) and probably depend on different factors; Ct-B-Ar might be due to the high mechanical load acting on the skeletal region, whereas endosteal MAR likely depends on the bone modeling processes that lead to new bone deposition at the endosteal level and bone resorption at the periosteal one.

As far as serum parameters are concerned, it is not surprising to observe after one month of experiment that Ca and P levels are similar in all groups, as a consequence of the early bone response by means of mineral homeostasis. Also serum mean values of OPG and BALP are similar in all groups, because in all groups bone deposition due to skeletal growth is not altered by diet, in particular in cortical bone. Concerning CrossLaps and PTH (1-84), the excessive individual variability among the animals inside the same group did not allow understanding and discussing the data obtained.

In conclusion, the relevance of this paper performed on the rat model lies in the detailed documentation of the fact that the lack of calcium in the diet does not lead to a unique bone answer. It is to be underlined in fact that the various answers recorded in the different sites of bony architectures pertaining to specific skeletal segments are due to the different main involvement of each skeletal region in maintaining mineral or skeletal homeostasis. As a consequence, the bone modeling processes are differently affected in answering induced biochemical osteoporosis. Regardless of the main topic of the present paper, data here reported on PTH (1-34) seems to indicate that Teriparatide, used with good results as therapeutic support in* recovering* bone fragility [39–42], does not display* preventive* effects, since no significant differences were found between the two groups of animals fed calcium-deprived diet with/without the drug administration.

The present investigation based on animal models represents a good starting point for successive studies on bone alterations during unbalanced calcium metabolism, as frequently occurring in aging, with the aim of studying in detail timing and manners of evolution and recovery in human biochemical osteoporosis with/without administration of PTH (1-34). In particular, the attention will be focused on the type of osteogenesis of the newly formed bone during bone mass recovering that, as previously demonstrated [43, 44], can occur in two different manners (*static* and* dynamic* osteogenesis) and imply different bone quality by the mechanical viewpoint.

## Figures and Tables

**Table 1:** Body weights of rat at the time of arrival and sacrifice.

Group	Arrival weight	Sacrifice weight
1	406 ± 48	426 ± 48
2	405 ± 32	526 ± 47
3	405 ± 32	528 ± 70
4	397 ± 25	504 ± 40

All values are expressed as mean ± sd, ANOVA followed by Bonferroni's test. Group 1: baseline; Group 2: control, normal diet; Group 3: calcium-deprived diet for 4 weeks; Group 4: calcium-deprived diet, plus concomitant administration of PTH (1-34) 40 *µ*g/kg/day, for 4 weeks.

**Table 2:** Static histomorphometric parameters of trabecular bone in L5 vertebral body sections.

Group	BV/TV (%)	Tb.Th (*µ*m)	Tb.N (*n*/mm)	Tb.Sp (*µ*m)
1	24.54 ± 2.28	59.56 ± 3.14	4.14 ± 0.54	185.12 ± 28.70
2	23.35 ± 2.31	53.29 ± 2.28	4.39 ± 0.52	180.33 ± 26.25
3	14.97 ± 3.55^∗∗,##^	50.75 ± 6.50	2.93 ± 0.41^∗,##^	298.79 ± 53.81^∗∗,##^
4	14.36 ± 0.73^∗∗,##^	47.07 ± 2.12^∗^	3.03 ± 0.06^∗,##^	285.34 ± 11.12^∗,##^

All values are expressed as mean ± sd, ANOVA followed by Bonferroni's test: ^∗^ *P* < 0.05, ^∗∗^ *P* < 0.01 versus Group 1; ^##^ *P* < 0.001 versus Group 2. Group 1: baseline; Group 2: control, normal diet; Group 3: calcium-deprived diet for 4 weeks; Group 4: calcium-deprived diet, plus concomitant administration of PTH (1-34) 40 *µ*g/kg/day, for 4 weeks.

**Table 3:** Static histomorphometric parameters of cortical bone in L5 vertebral body sections.

Group	Anterolateral Ct.Th	Posterior Ct.Th
1	311.84 ± 71.47	176.15 ± 33.10
2	321.49 ± 35.97	168.85 ± 26.67
3	197.84 ± 9.54^∗∗∗,###^	148.23 ± 35.69
4	162.67 ± 10.53^∗∗∗,###^	130.37 ± 13.37

All values (*µ*m) are expressed as mean ± sd, ANOVA followed by Bonferroni's test: ^∗∗∗^ *P* < 0.001 versus Group 1; ^###^ *P* < 0.001 versus Group 2. Group 1: baseline; Group 2: control, normal diet; Group 3: calcium-deprived diet for 4 weeks; Group 4: calcium-deprived diet, plus concomitant administration of PTH (1-34) 40 *µ*g/kg/day, for 4 weeks.

**Table 4:** Dynamic histomorphometric parameters in L5 vertebral body sections.

Group	Anterior Ct Nf B Ar (mm^2^)	Anterior Ct MAR (*µ*m/day)	Posterior Ct Nf B Ar (mm^2^)	Posterior Ct MAR (*µ*m/day)	Tb MS (%)
2	0.063 ± 0.08	4.10 ± 0.71	0.035 ± 0.01	3.47 ± 0.50	11.40 ± 2.30
3	0.075 ± 0.04	4.19 ± 1.40	0.033 ± 0.02	3.26 ± 0.66	53.03 ± 6.44^###^
4	0.076 ± 0.01	4.22 ± 0.39	0.036 ± 0.07	3.42 ± 0.54	50.80 ± 9.52^###^

All values are expressed as mean ± sd, ANOVA followed by Bonferroni's test: ^###^ *P* < 0.001 versus Group 2. Group 2: control, normal diet; Group 3: calcium-deprived diet for 4 weeks; Group 4: calcium-deprived diet, plus concomitant administration of PTH (1-34) 40 *µ*g/kg/day, for 4 weeks.

**Table 5:** Static histomorphometric parameters in middiaphyseal femoral sections.

Group	Round-shaped sections			Sharp-edge-shaped sections		
	Total cross section area	Cortical bone area	Medullary canal area	Total cross section area	Cortical bone area	Medullary canal area
1	11.07 ± 0.61	6.61 ± 0.44	4.36 ± 0.57	10.94 ± 0.50	6.74 ± 0.48	4.19 ± 0.55
2	12.19 ± 1.38	7.35 ± 0.84	4.83 ± 0.54	12.43 ± 1.50	7.73 ± 0.88	4.70 ± 0.53
3	12.71 ± 0.75	6.90 ± 0.48	5.64 ± 0.38^∗^	12.97 ± 0.87	7.25 ± 0.68	5.67 ± 0.32^∗#^
4	11.71 ± 0.78	6.50 ± 0.35	5.17 ± 0.49	12.20 ± 0.89	6.67 ± 0.45	5.50 ± 0.46^∗^

All values (mm^2^) are expressed as mean ± sd, ANOVA followed by Bonferroni's test: ^∗^ *P* < 0.05 versus Group 1; ^#^ *P* < 0.05 versus Group 2. Group 1: baseline; Group 2: control, normal diet; Group 3: calcium-deprived diet for 4 weeks; Group 4: calcium-deprived diet, plus concomitant administration of PTH (1-34) 40 *µ*g/kg/day, for 4 weeks.

**Table 6:** Dynamic histomorphometric parameters (Nf B Ar-MAR-MS) in middiaphyseal femoral sections.

Group	Round-shaped sections		Sharp-edge-shaped sections	
	Periosteal Nf B Ar (mm^2^)	Endosteal Nf B Ar (mm^2^)	Periosteal Nf B Ar (mm^2^)	Endosteal Nf B Ar (mm^2^)
2	0.27 ± 0.10	0.18 ± 0.15	0.82 ± 0.23	0.05 ± 0.06
3	0.23 ± 0.09	0.14 ± 0.09	0.77 ± 0.34	0.001 ± 0.001
4	0.20 ± 0.10	0.06 ± 0.04	0.80 ± 0.34	0.005 ± 0.012

	Periosteal MAR (*µ*m/day)	Endosteal MAR (*µ*m/day)	Periosteal MAR (*µ*m/day)	Endosteal MAR (*µ*m/day)

2	2.38 ± 0.49	1.68 ± 0.92	3.67 ± 0.70	1.09 ± 0.99
3	2.82 ± 1.26	2.25 ± 0.55	3.65 ± 1.13	0.14 ± 0.32
4	2.56 ± 0.59	2.03 ± 1.09	3.84 ± 1.27	0.17 ± 0.37

	Periosteal MS (%)	Endosteal MS (%)	Periosteal MS (%)	Endosteal MS (%)

2	52.40 ± 12.91	41.78 ± 18.95	67.39 ± 18.58	29.25 ± 19.06
3	54.89 ± 12.62	42.77 ± 21.83	84.45 ± 10.06	49.94 ± 16.52
4	62.81 ± 14.60	41.81 ± 5.19	72.26 ± 20.26	41.77 ± 7.07

All values are expressed as mean ± sd, ANOVA followed by Bonferroni's test. Group 1: baseline; Group 2: control, normal diet; Group 3: calcium-deprived diet for 4 weeks; Group 4: calcium-deprived diet, plus concomitant administration of PTH (1-34) 40 *µ*g/kg/day, for 4 weeks.

**Table 7:** Static histomorphometric parameters in distal metaphyseal femoral sections.

Group	BV/TV (%)	Tb.Th (*µ*m)	Tb.N (*n*/mm)	Tb.Sp (*µ*m)	Ct-B-Ar (mm^2^)
1	18.26 ± 7.36	51.63 ± 10.19	3.16 ± 0.67	255.36 ± 104.87	6.01 ± 0.41
2	17.76 ± 4.78	50.36 ± 7.92	3.49 ± 0.51	241.08 ± 49.04	5.93 ± 0.70
3	6.94 ± 1.47^∗#^	42.88 ± 5.01	1.62 ± 0.40^∗∗###^	601.63 ± 165.23^∗∗##^	5.71 ± 0.19
4	7.50 ± 2.05^∗#^	43.89 ± 8.01	1.65 ± 0.19^∗∗###^	564.60 ± 84.57^∗##^	5.91 ± 0.60

All values are expressed as mean ± sd, ANOVA followed by Bonferroni's test: ^∗^ *P* < 0.05, ^∗∗^ *P* < 0.01 versus Group 1; ^#^ *P* < 0.05, ^##^ *P* < 0.01, and ^###^ *P* < 0.001 versus Group 2. Group 1: baseline; Group 2: control, normal diet; Group 3: calcium-deprived diet for 4 weeks; Group 4: calcium-deprived diet, plus concomitant administration of PTH (1-34) 40 *µ*g/kg/day, for 4 weeks.

**Table 8:** Dynamic histomorphometric parameters in distal metaphyseal femoral sections.

Group	Endosteal MAR (*µ*m/day)	Tb MS (%)
2	2.91 ± 0.45	34 ± 7.88
3	2.87 ± 0.11	48 ± 4.55
4	2.81 ± 0.38	50.5 ± 7.23^#^

All values are expressed as mean ± sd, ANOVA followed by Bonferroni's test: ^#^ *P* < 0.05 versus Group 2. Group 2: control, normal diet; Group 3: calcium-deprived diet for 4 weeks; Group 4: calcium-deprived diet, plus concomitant administration of PTH (1-34) 40 *µ*g/kg/day, for 4 weeks.

**Table 9:** Values of serum levels at the end of the experiments: Ca, P, OPG, BALP, CrossLaps, and PTH (1-84).

Group	Ca mg/dL	P mg/dL	OPG ng/ml	BALP ng/ml	CrossLaps ng/mL	PTH (1-84) pg/ml
1	10.02	7.38	0.75	7.12	38.67	41.24
	10.42	5.87	0.67	7.22	59.76	40.65
	10.34	7.63	0.72	6.78	73.86	33.59

Mean value ± sd	10.26 ± 0.21	6.96 ± 0.95	0.71 ± 0.04	7.04 ± 0.23	57.40 ± 17.71	38.49 ± 4.25

2	9.89	6.67	0.75	6.53	36.62	45.35
	10.14	6.71	0.65	6.94	40.61	80.06
	9.46	8.13	0.76	6.51	45.31	84.18
	9.48	5.75	0.74	8.00	84.52	31.24
	9.75	6.00	0.68	6.55	52.53	31.82

Mean value ± sd	9.74 ± 0.28	6.65 ± 0.92	0.71 ± 0.04	6.90 ± 0.63	51.91 ± 19.16	54.52 ± 25.85

3	9.78	7.00	0.69	6.74	52.77	61.82
	10.10	6.55	0.60	6.33	86.80	79.47
	10.18	8.78	0.67	7.28	58.20	98.29
	9.90	6.86	0.57	6.59	83.06	39.47
	10.11	7.57	0.85	7.26	51.01	115.35

Mean value ± sd	10.01 ± 0.16	7.35 ± 0.87	0.67 ± 0.10	6.84 ± 0.41	66.36 ± 17.20	78.88 ± 29.79

4	10.36	6.97	0.70	6.82	120.33	80.06
	10.52	5.96	0.67	6.33	38.04	72.41
	9.86	7.35	0.76	7.00	85.78	41.24
	10.00	7.39	0.62	6.29	62.14	90.65
	10.11	7.46	0.71	6.90	47.00	45.35

Mean value ± sd	10.12 ± 0.28	7.04 ± 0.72	0.69 ± 0.05	6.63 ± 0.37	58.24 ± 20.88	62.41 ± 23.35

ANOVA followed by Bonferroni's test. Group 1: baseline; Group 2: control, normal diet; Group 3: calcium-deprived diet for 4 weeks; Group 4: calcium-deprived diet, plus concomitant administration of PTH (1-34) 40 *µ*g/kg/day, for 4 weeks.
